# A Manual of Pathology

**Published:** 1884-01

**Authors:** 


**A Manual of Pathology.** By JOSEPH COATS, M. D., Pathologist to the Western Infirmary and the Sick Children's Hospital, Glasgow, Lecturer on Pathology in the Western Infirmary, President of the Pathological and Clinical Society of Glasgow. With three hundred and thirty-nine illustrations. Philadelphia: H. C. Lea's Son & Co. 1883.

The number of new books and new editions of old books on pathology which have lately appeared is an evidence of the increased interest in and study of this important branch of medical knowledge. The present work is one of the most valuable in any language, and may fairly be called a complete systematic treatise; and as the distinguished author has for years been engaged as a practical pathologist, and in teaching large classes of students, he has succeeded in making the work not only thorough, but practical in its scope. For this reason it is peculiarly valuable to the general practitioner. We believe that it will take the first place in America, as it has in England, among the works upon this subject. The illustrations are excellent.

